# Chikungunya Fever Outbreak, Bhutan, 2012

**DOI:** 10.3201/eid1910.130453

**Published:** 2013-10

**Authors:** Sonam Wangchuk, Piyawan Chinnawirotpisan, Tshering Dorji, Tashi Tobgay, Tandin Dorji, In-Kyu Yoon, Stefan Fernandez

**Affiliations:** Ministry of Health, Thimphu, Bhutan (S. Wangchuk, T. Dorji, T. Tobgay, T. Dorji);; Armed Forces Research Institute of Medical Sciences, Bangkok, Thailand (P. Chinnawirotpisan, I.-K. Yoon, S. Fernandez)

**Keywords:** Chikungunya fever, Chikungunya virus, outbreak, Bhutan, Togaviridae, Alphavirus, viruses, Aedes aegypti, Aedes albopictus, re-emerging disease

## Abstract

In 2012, chikungunya virus (CHIKV) was reported for the first time in Bhutan. IgM ELISA results were positive for 36/210 patient samples; PCR was positive for 32/81. Phylogenetic analyses confirmed that Bhutan CHIKV belongs to the East/Central/South African genotype. Appropriate responses to future outbreaks require a system of surveillance and improved laboratory capacity.

Chikungunya fever is caused by infection with chikungunya virus (CHIKV; family *Togaviridae*, genus *Alphavirus*). CHIKV was first isolated from humans and mosquitoes during an epidemic of chikungunya fever in Newala, Tanzania, during 1952–1953 (*1*). The virus is transmitted to humans primarily by *Aedes aegypti* and *Ae.* *albopictus* mosquitoes. Chikungunya fever can cause lingering joint pain, often lasting several weeks; the disease is rarely life-threatening and, unlike dengue virus infection, does not cause severe hemorrhagic manifestations or shock (*2*). Outbreaks of chikungunya fever are sporadic, and there is no licensed vaccine to protect against the disease.

In addition to the chikungunya fever epidemic in Tanzania in 1952–1953, several other outbreaks have affected millions of persons in eastern, western, and central Africa and Asia. The first of many chikungunya fever outbreaks in Southeast Asia was reported in Bangkok, Thailand, in 1958, followed by outbreaks in Sri Lanka; Kolkata and Chennai, India; and elsewhere (*3**–**6*). CHIKV reemergence in Indonesia and India caused epidemics during 1999–2003 and 2005–2006, respectively; 1.3 million cases of chikungunya fever occurred in India during the epidemics (*6*,*7*). In 2004, a chikungunya fever outbreak occurred in the Indian Ocean region. On Réunion Island, 266,000 cases, affecting ≈40% of the population, and ≈250 deaths were recorded (*7*). We report details of a chikungunya fever outbreak in Bhutan in July 2012; CHIKV had not been reported in Bhutan before this outbreak.

## The Study

In July 2012, the Public Health Laboratory (PHL), Department of Public Health, Ministry of Health in Thimphu, Bhutan, received reports of suspected chikungunya fever cases. A total of 215 acute-phase serum samples were obtained from patients at hospitals in the southwestern districts of Samtse, Chukha, and Thimphu (Technical Appendix Figure) and sent to PHL for testing (Table 1) (*8*). Samples were maintained at 4°C until arrival at PHL. Most patients from whom a serum sample was obtained were inpatients 15–44 years of age. Of the 215 samples, 84 (39%) were from female patients (mean age of 27.2 years, range 4 months to 68 years, and 131 (61%) were from male patients (mean age 31.5 years, range 2–78 years). 

**Table 1:** IgM ELISA results and signs and symptoms for patients with suspected chikungunya fever, Bhutan, 2012

District, patient sex	No. patients	Mean age, y	No. positive samples/no. tested (%)	No. patients for whom medical history obtained	No. (%) patients with sign/symptom			
					Fever	Arthralgia	Headache	Rash
Chuka								
F	21	19.6	2/21 (9.5)	3	3 (100)	1 (33)	0	0
M	28	28.8	8/27 (29.6)	5	5 (100)	2 (40)	3 (60)	0
Samtse								
F	40	28.2	3/39 (7.7)	25	25 (100)	14 (56)	19 (76)	3 (12)
M	50	28.2	7/47 (14.9)	30	30 (100)	16 (53)	20 (67)	1 (3)
Thimphu								
F	23	32.4	6/23 (26.1)	2	2 (100)	2 (100)	2 (100)	0
M	53	36.1	10/53 (18.9)	14	14 (100)	13 (93)	11 (79)	2 (14)
Total	215	–	36/210 (17)	79	79 (100)	48 (60.1)	55 (69.6)	6 (7.6)

Serum samples from 210 of the 215 patients were tested for CHIKV IgM by ELISA (Table 1). The CHIKV ELISA was developed by the National Institute of Virology in Pune, India, and supplied to PHL by the World Health Organization. Of the 210 samples, 36 (17.1%) were positive for CHIKV IgM. As determined by Fisher exact test, there were no significant differences in the percentage of IgM ELISA–positive cases in the 3 districts (p>0.2) or for male versus female patients (p>0.08). Medical records were provided for only 79 patients; significantly fewer records were provided for patients in Chuka than for patients in Samtse and Thimphu (p>0.003). Of the 79 patients with medical records, all had a fever, 48 (60.1%) had arthralgia, 55 (69.6%) had headache, and 6 (7.6%) had a rash (*8*). There were no substantial differences in the distribution of symptoms by patient sex of district of residence. 

The first 78 (37%) acute-phase serum samples received by PHL were shipped frozen to the Armed Forces Research Institute of Medical Sciences (AFRIMS) in Bangkok, Thailand, for testing by nested reverse transcription PCR (RT-PCR) amplification of the capsid gene (Table 2) (*9*). Of these 78 samples, 32 (41%) were positive for CHIKV. The difference in distribution of positive and negative RT-PCR results by patient sex was not statistically significant in any of the 3 districts (p>0.1, Fisher exact test). Of 8 IgM ELISA–positive samples sent to AFRIMS for testing, 4 (50%) were positive by RT-PCR, and of 67 IgM ELISA–negative samples sent to AFRIMS for testing, 27 (40.3%) were positive (Table 2). 

**Table 2:** IgM ELISA and PCR results for 78 patients with suspected chikungunya fever, Bhutan, 2012*

District, patient sex, no. patients	IgM ELISA†				Nested reverse transcription PCR‡	
	No. positive/no. tested (%)	No. negative/no. tested (%)	No. not tested/no. total (%)		No. positive/no. tested (%)	No. negative/no. tested (%)
Chuka§						
F, n = 6	0/6				NA	NA
		6/6 (100)			2/6 (33)	4/6 (67)
M, n = 10	0/10				NA	NA
		10/10 (100)			3/10 (30)	7/10 (70)
Samtse¶						
F, n = 25	1/24 (4)				0/1	1/1 (100)
		23/24 (96)			12/23 (52)	11/23 (48)
			1/25 (4)		1/1 (100)	0/1
M, n = 34	6/32 (19)				4/6 (67)	2/6 (33)
		26/32 (81)			9/26 (35)	17/26 (65)
			2/34 (6)		0/2	2/2 (100)
Thimphu‖						
F, n = 1	0/1				NA	NA
		1/1 (100)			0/1	1/1 (100)
M, n = 2	1/2 (50)				0/1	1/1 (100)
		1/2 (50)			1/1 (100)	0/1
Total	8/75 (11)	67/75 (89)	3/59 (5)		32/78 (41)	46/78 (59)

*NA, not applicable. †Results reported by the Public Health Laboratory, Department of Public Health, Ministry of Health, Thimphu, Bhutan. ‡Results reported by the Armed Forces Research Institute of Medical Sciences, Bangkok, Thailand. §Mean age for female patients was 28.1 y; mean age for male patients was 26.5 y. ¶Mean age for female patients was 28.8 y; mean age for male patients was 28.7 y. ‖Mean age for female patients was not reported; mean age for male patients was 31 y.

The first RT-PCR–confirmed CHIKV-positive specimen was collected on July 12, 2012, from a patient for whom the date of illness onset was listed as July 11. On July 9, the patient had traveled through West Bengal, India, en route from Thimphu to Samtse, Bhutan. All confirmed chikungunya fever case-patients in Thimphu had traveled to Phuentsholing, Bhutan, or other places in southern Bhutan affected by the outbreak.

**Figure 1:**
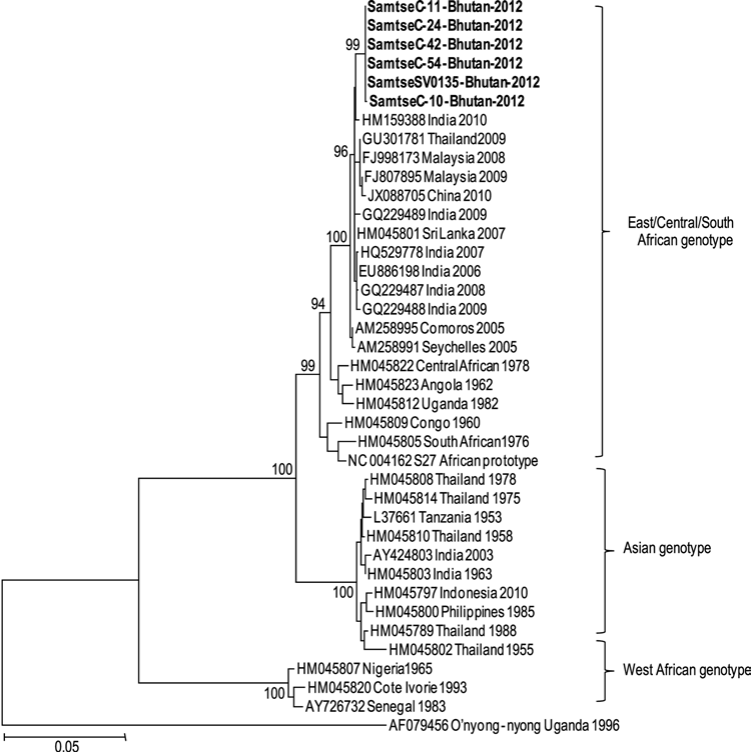
Phylogenetic analyses of the envelope protein 1 (E1) sequences of chikungunya virus (CHIKV) isolated in Bhutan in 2012. The neighbor-joining tree was constructed by using MEGA 5.0 (www.megasoftware.net); bootstrap values were obtained from 2,000 replicates. The unrooted tree topology was based on multiple alignments of the E1 gene nucleotide sequences (1,320 bp) and 34 CHIKV E1 regions, representing a wide range of localities, from GenBank. Each CHIKV isolate is represented by the country of origin, year of isolation, and GenBank accession number. Six representative isolates from the 2012 outbreak are included; the isolates show 99.9%–100% nt identity with each other and 99.4%–99.5% nt identity with circulating Indian 2012 CHIKV (GenBank accession no. HM159388). Scale bar indicates nucleotide substitutions per site.

**Figure 2:**
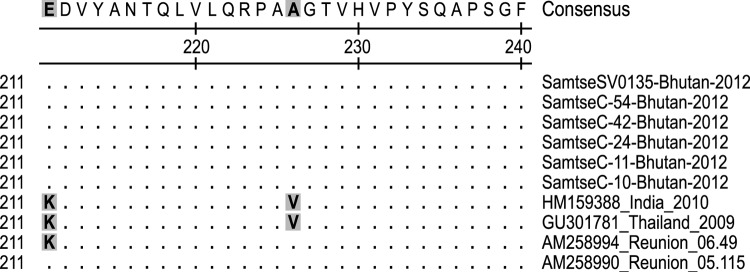
Amino acids at positions 211 and 226 of the chikungunya virus (CHIKV) envelope protein 1 (E1) and alignment of aa 211–240 of Bhutan 2012 CHIKV isolates with Réunion Island 2005, Indian 2010, and Thailand 2009 CHIKV isolates. Gray shading indicates residues 211 and 226 of the E1 protein.

Six specimens, which were selected on the basis of their relatively strong CHIKV RT-PCR results, were used for partial sequencing of the envelope protein 1 (E1) gene; all 6 specimens were from Samtse. The 1,320-bp sequences were compared against 34 other GenBank E1 sequences from different localities (Figure 1). The 6 representative isolates (GenBank accession nos. KC731581–KC731586) were 99.9%–100% identical at the nucleotide level and 99.8% identical at the amino acid level. Phylogenetic analyses of the partial E1 sequences showed that the 2012 outbreak of chikungunya fever in Bhutan was caused by a CHIKV of the East/Central/South African genotype, and the virus was removed only by 0.5% from HM159388 India 2010 CHIKV, 0.7% from GQ229488 India 2009 CHIKV, and 0.5% from HM045801 Sri Lanka 2007 CHIKV. Amino acid sequence analyses showed that all 6 Bhutan isolates lack the E1 gene mutations, which led to the A226V E1 protein change found in the Réunion Island 06.49 strain and which are closely associated with a higher transmission rate (Figure 2) (*10*). In addition, the 2012 Bhutan strain still retained the E211 amino acid, which is also found in the India 2010 strain (*11*).

## Conclusions

The results of our phylogenetic analyses suggest that the Bhutan CHIKV originated from CHIKV found in India. This finding is not surprising given the proximity of the 2 countries and the expanding range of the virus. The 2012 chikungunya fever outbreak in Bhutan occurred in the same area as a 2004 outbreak of dengue virus infection (*12*); this area shares borders with West Bengal and Assam, India. 

The IgM ELISA and RT-PCR results in our study did not always agree; however, the discrepancies may be explained by the time of collection relative to onset of symptoms. Specimens collected early after illness onset were likely collected during the viremic state and thus positive by RT-PCR, but not by IgM ELISA. Specimens collected later after illness onset would likely have been IgM positive, even in the absence of detectable virus.

Because of several factors, the introduction of CHIKV virus into Bhutan was expected: the presence of the mosquito vectors (*Ae. aegypti* and *Ae.* *albopictus*) in southern districts, the increasing geographic reach of CHIKV, the predominance of a presumably naive population, urbanization and industrial growth in Bhutan, and the country’s unrestricted border with India. Another contributing factor may have been the circulation of a new East/Central/South African CHIKV strain, first isolated in 2005 from a patient on Réunion Island, that represents a distinct clade within the larger East/Central/South African phylogroup (*13*). This clade has the A226V mutation in the E1 protein, which leads to higher transmission and replication in *Ae. albopictus* mosquitoes (*10*). However, isolates collected during this outbreak lack this mutation and instead are more closely associated with the India 2010 strain (Figure 2).

The 2004 dengue virus infection outbreak (*12*) and now the 2012 chikungunya fever outbreak in Bhutan suggest that despite its remote location, the country is still vulnerable to epidemics and pandemics. A sound public health surveillance system and improved laboratory capacity is needed in Bhutan so that the country can appropriately respond to future disease outbreaks.

Technical Appendix Map of Bhutan showing areas involved in the 2012 outbreak of chikungunya fever.
