# Supplementary figures and images for: Chikungunya Fever Outbreak, Bhutan, 2012
Source: Emerg Infect Dis. 2013 Oct;19(10):1681–4. doi: 10.3201/eid1910.130453 (PMC3810753; doi:10.3201/eid1910.130453)

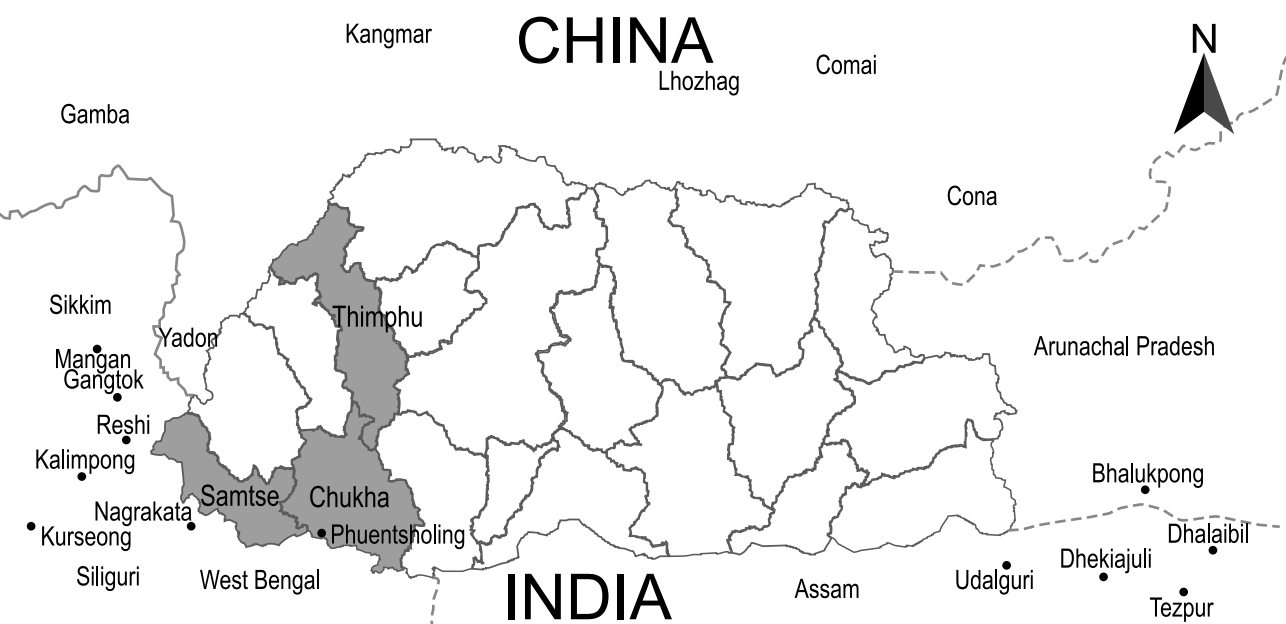

Supplement: Technical Appendix — Map of Bhutan showing areas involved in the 2012 outbreak of chikungunya fever. [file 13-0453-Techapp-s1.pdf]
